# Supplementary figures and images for: Mir221/222 drive synovial hyperplasia and arthritis by targeting cell cycle inhibitors and chromatin remodeling components
Source: eLife. 2024 Sep 5;13:e84698. doi: 10.7554/eLife.84698 (PMC11377061; doi:10.7554/eLife.84698)

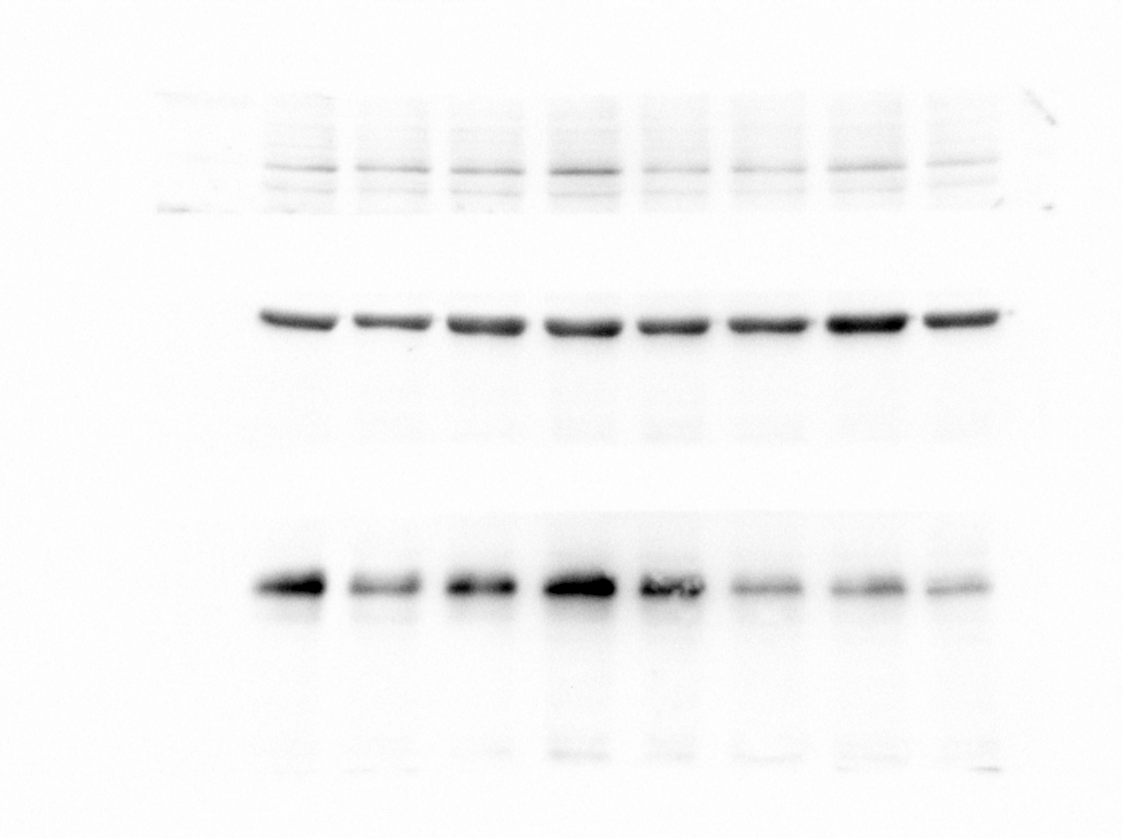

Supplement: Figure 4—source data 4. — Exposure set at 1 s for CDKN1B and ACTIN. [file elife-84698-fig4-data4.zip › Figure 4-Source data 4-raw-file.tif]

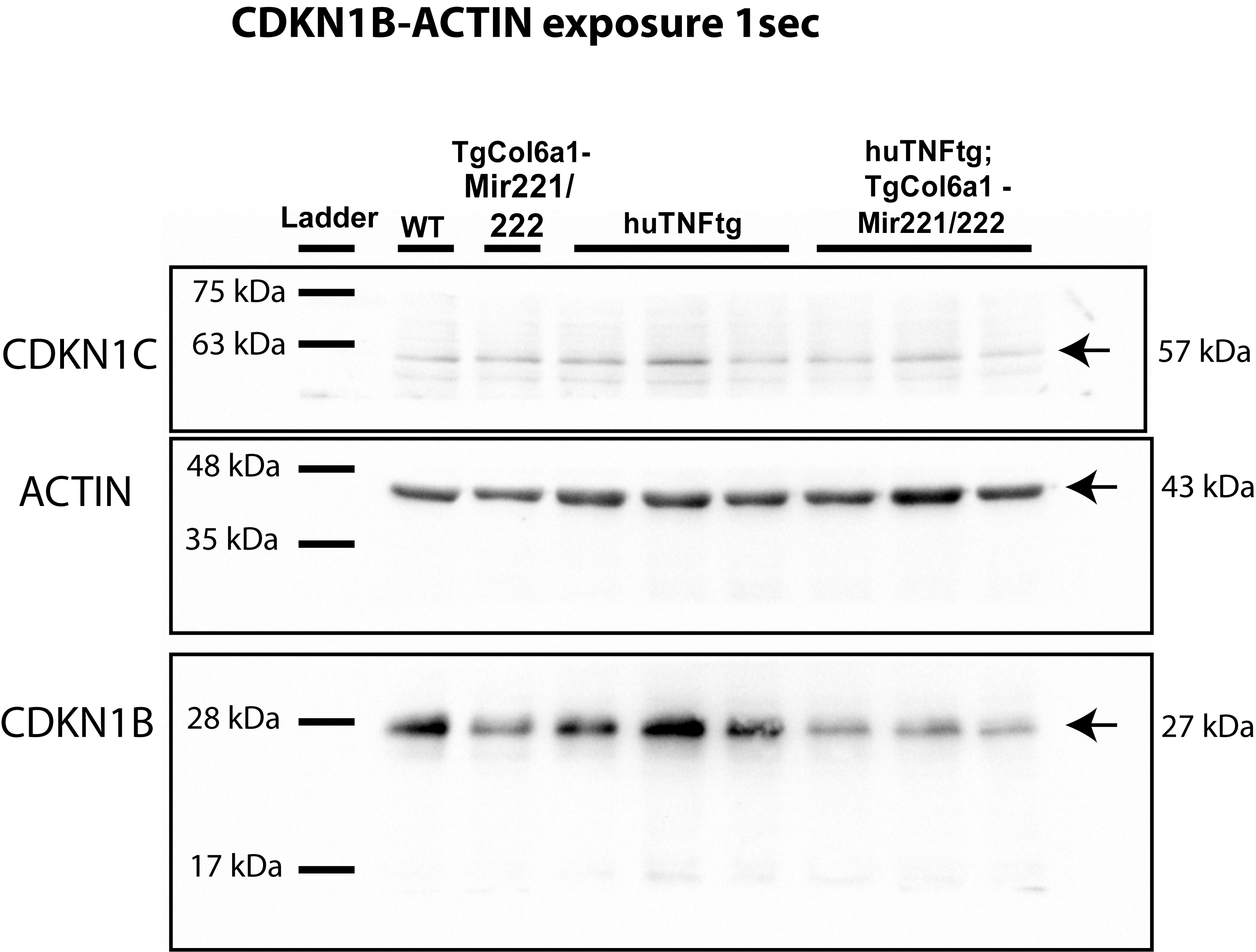

Supplement: Figure 4—source data 4. — Exposure set at 1 s for CDKN1B and ACTIN. [file elife-84698-fig4-data4.zip › Figure 4-Source data 4.tif]

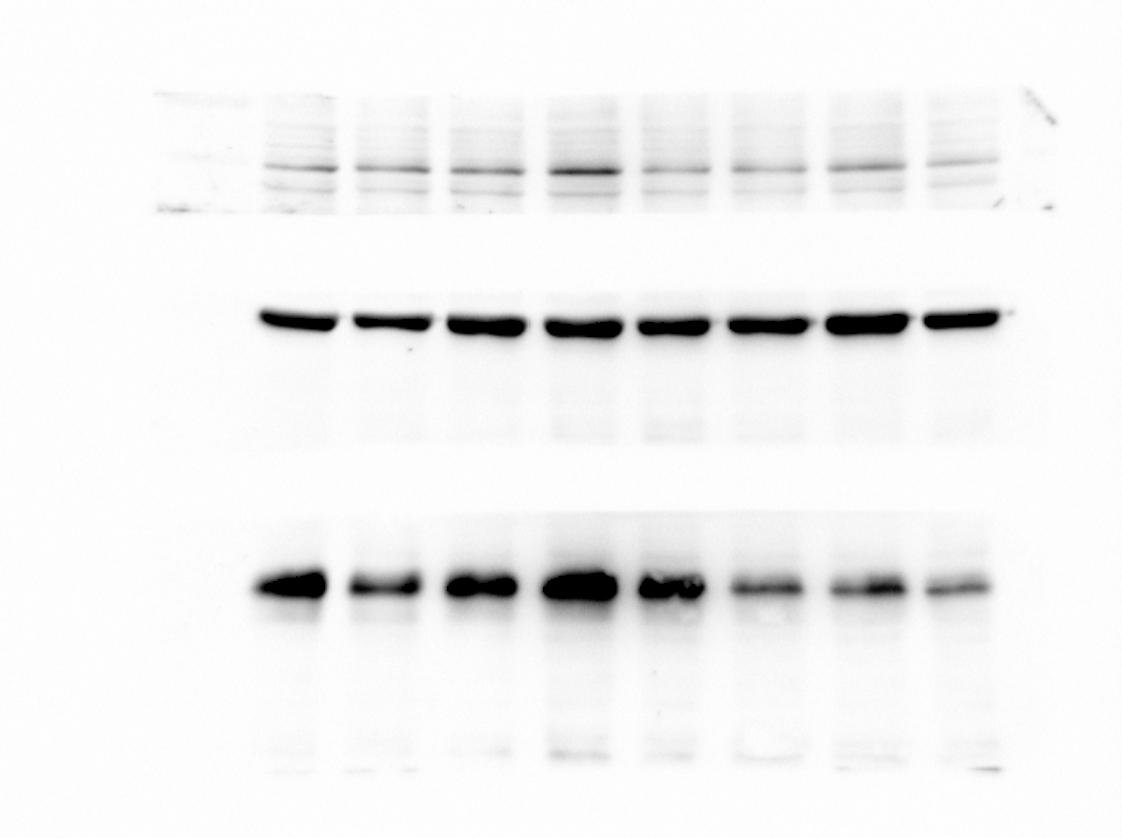

Supplement: Figure 4—source data 5. — Exposure set at 7 s for CDKN1C. [file elife-84698-fig4-data5.zip › Figure 4-Source data 5-raw-file.tif]

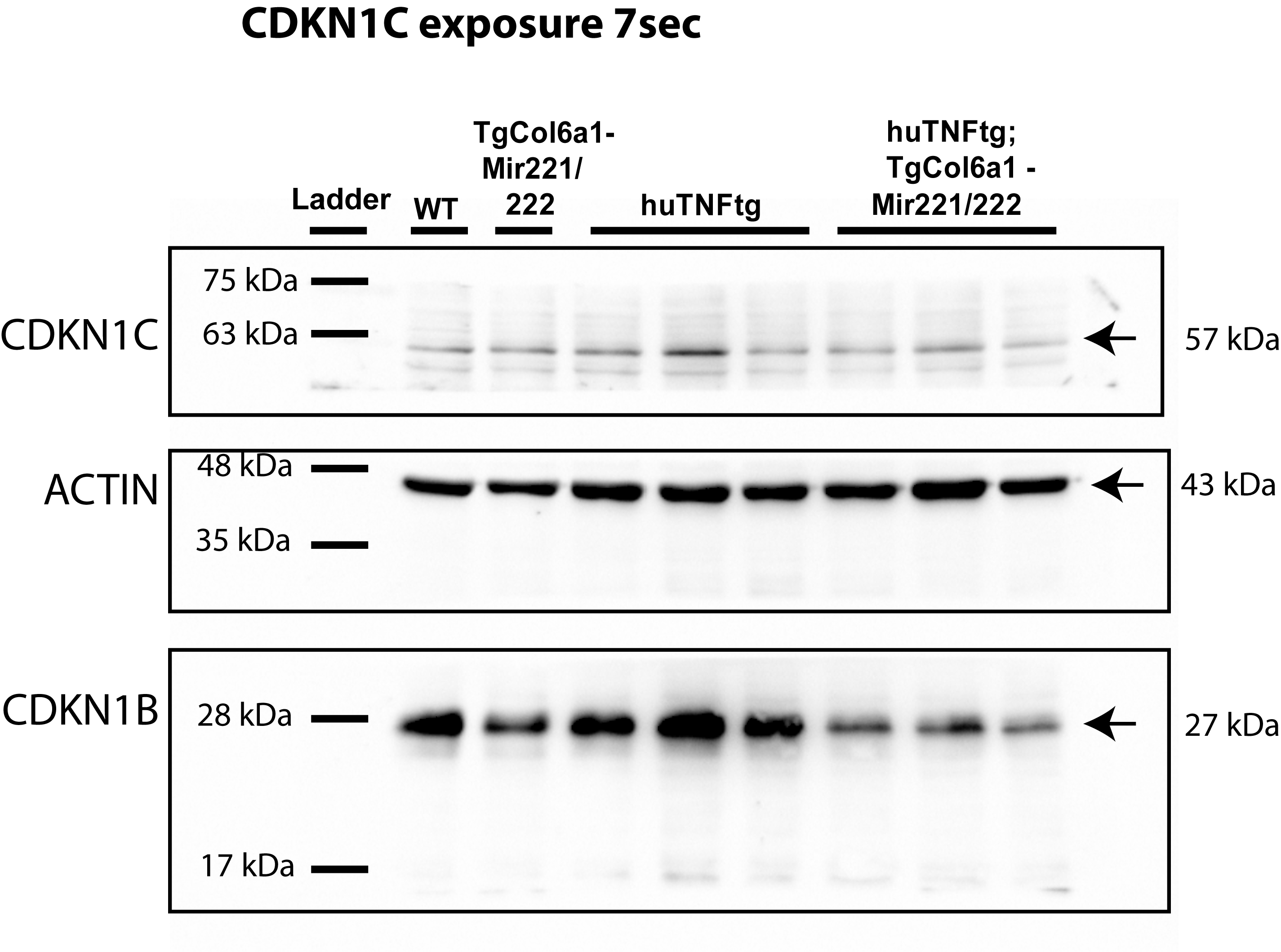

Supplement: Figure 4—source data 5. — Exposure set at 7 s for CDKN1C. [file elife-84698-fig4-data5.zip › Figure 4-Source data 5.tif]

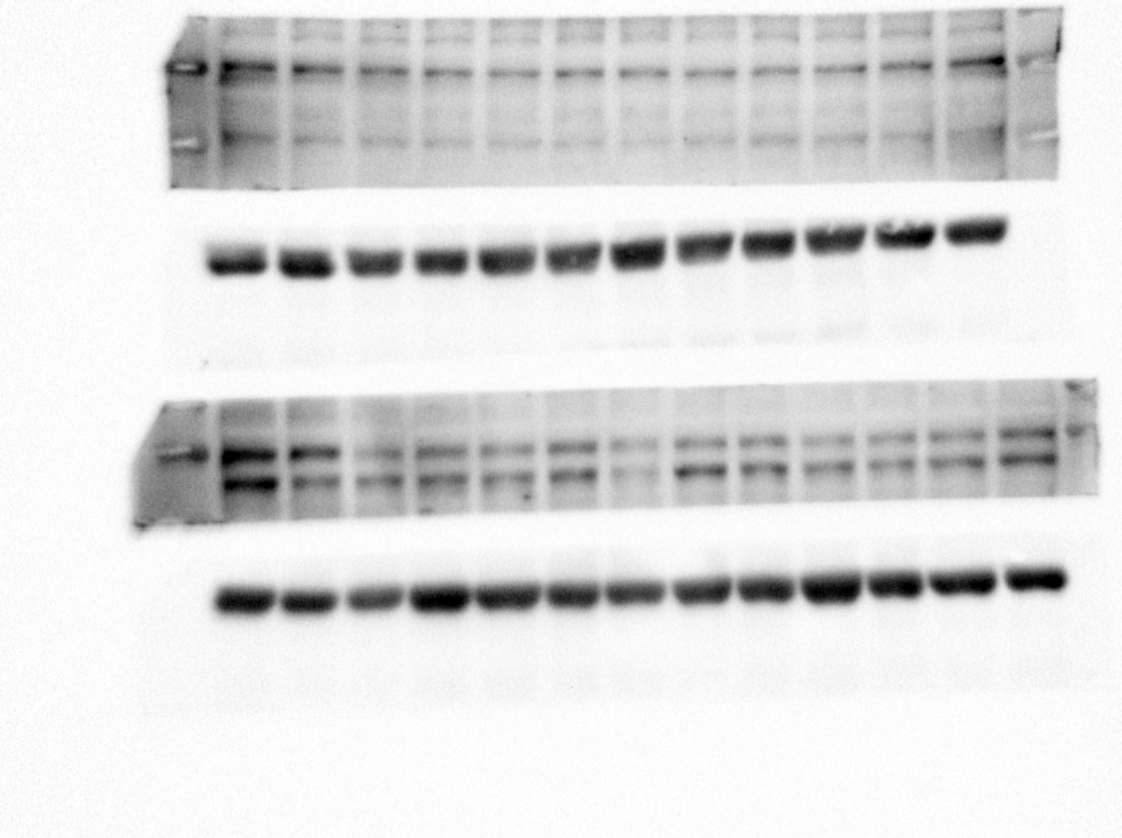

Supplement: Figure 4—source data 6. — Exposure set at 2 s for SMARCA1 and ACTIN. [file elife-84698-fig4-data6.zip › Figure 4-Source data 6-raw-file.tif]

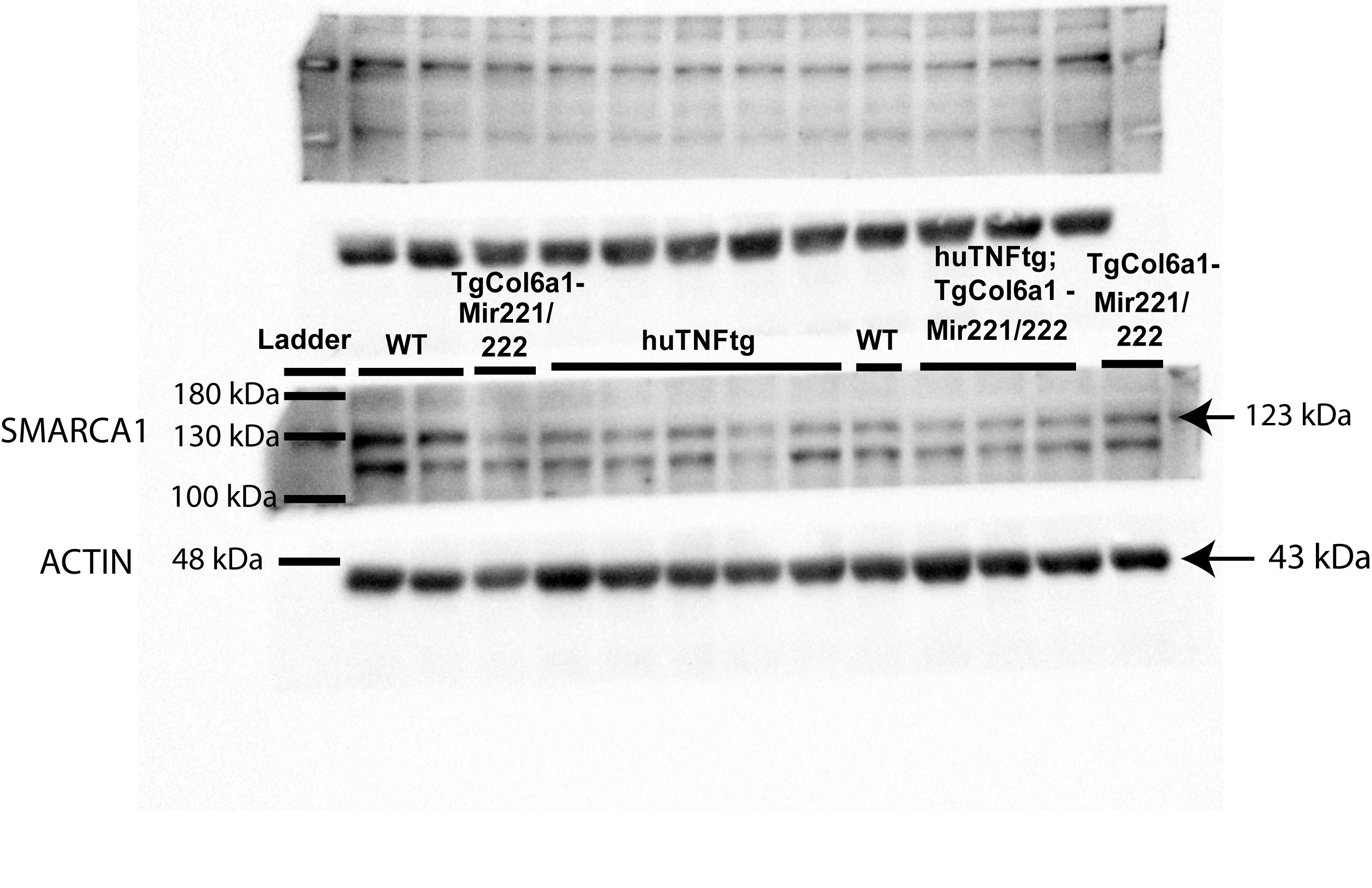

Supplement: Figure 4—source data 6. — Exposure set at 2 s for SMARCA1 and ACTIN. [file elife-84698-fig4-data6.zip › Figure 4-Source data 6.tif]

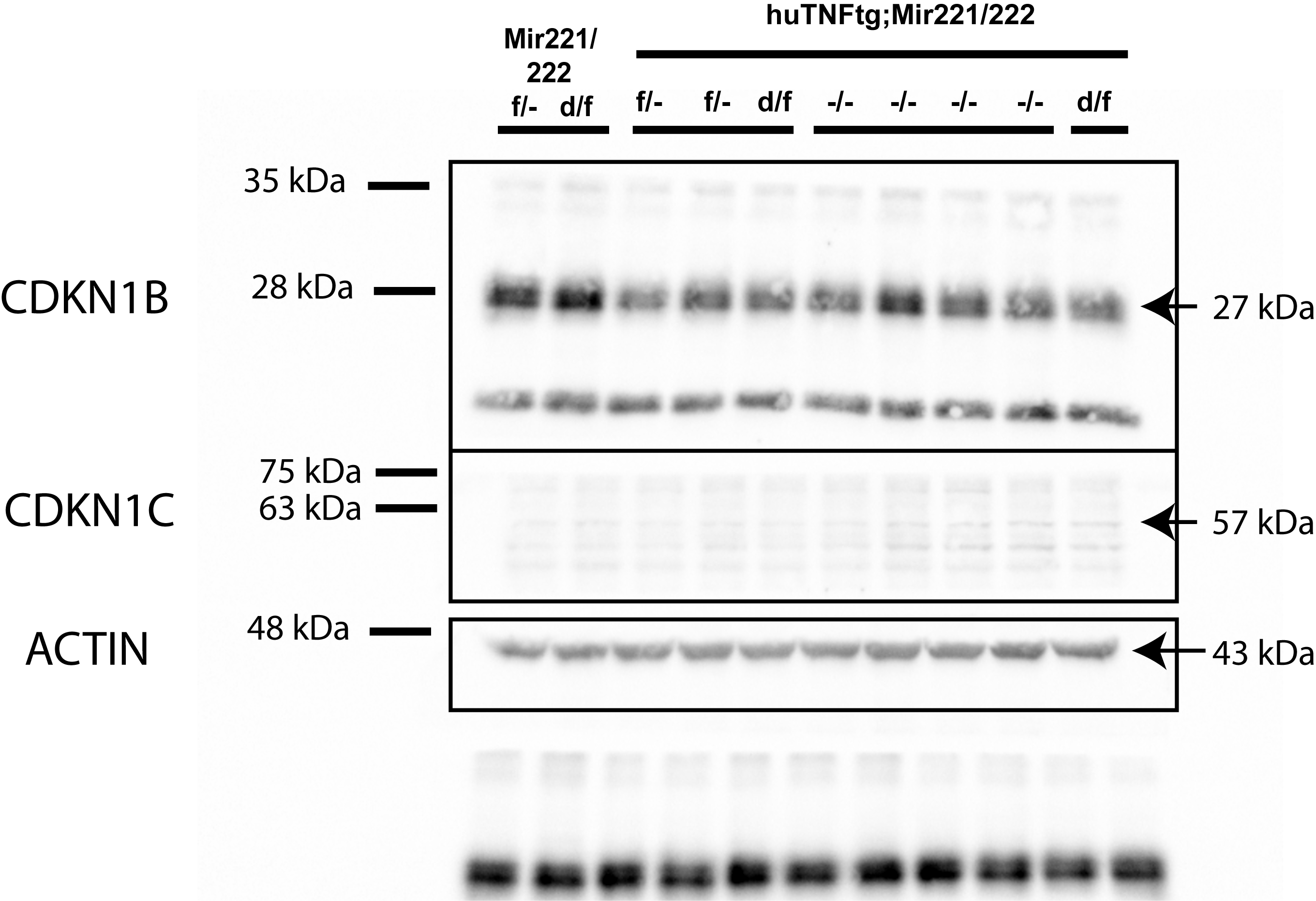

Supplement: Figure 5—source data 1. — Exposure set at 1 s for CDKN1B. [file elife-84698-fig5-data1.zip › Figure 5-Source data 1.tif]

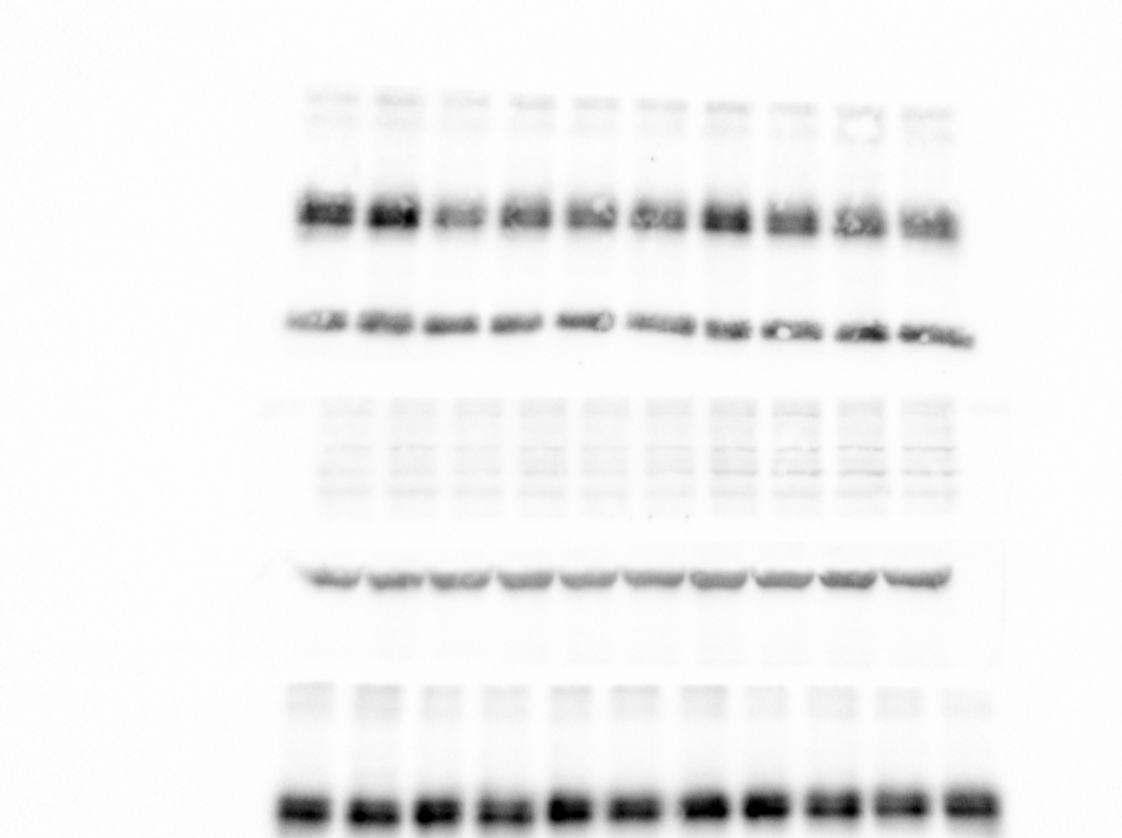

Supplement: Figure 5—source data 1. — Exposure set at 1 s for CDKN1B. [file elife-84698-fig5-data1.zip › Figure 5-Source data 1-raw-file.tif]

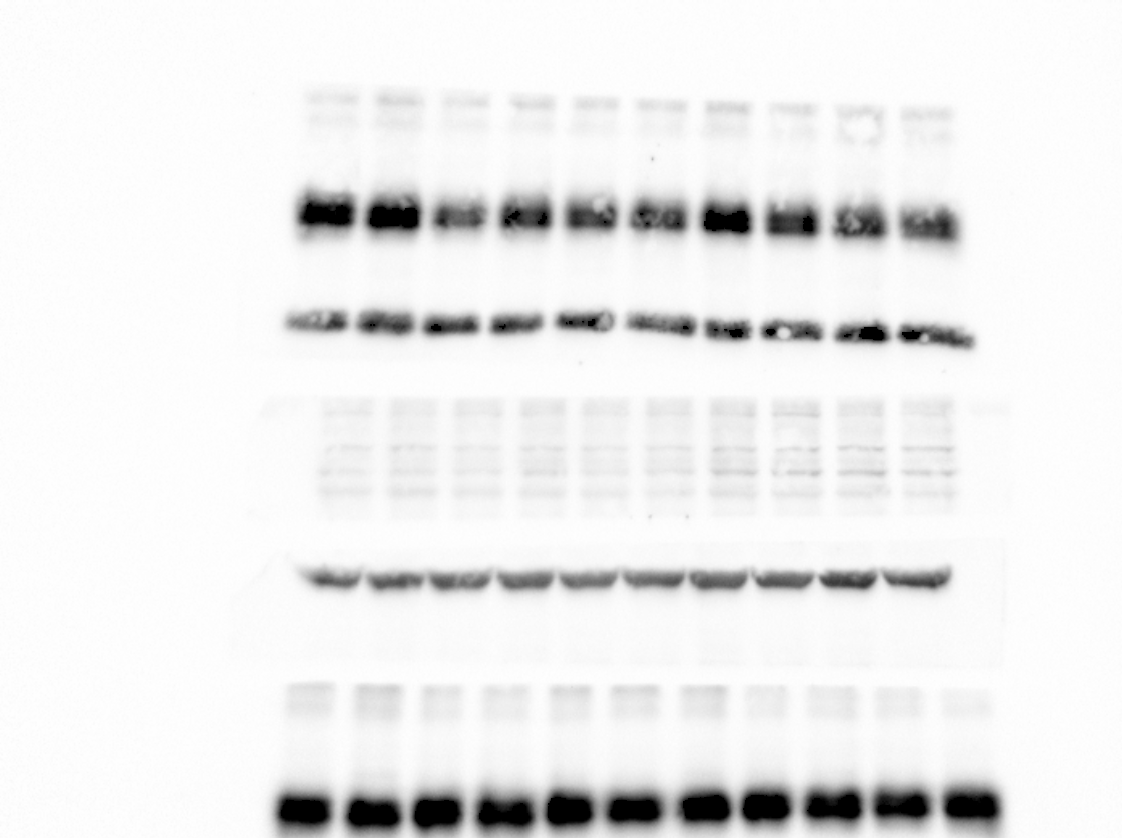

Supplement: Figure 5—source data 2. — Exposure set at 2.4 s for ACTIN. [file elife-84698-fig5-data2.zip › Figure 5-Source data 2-raw-file.tif]

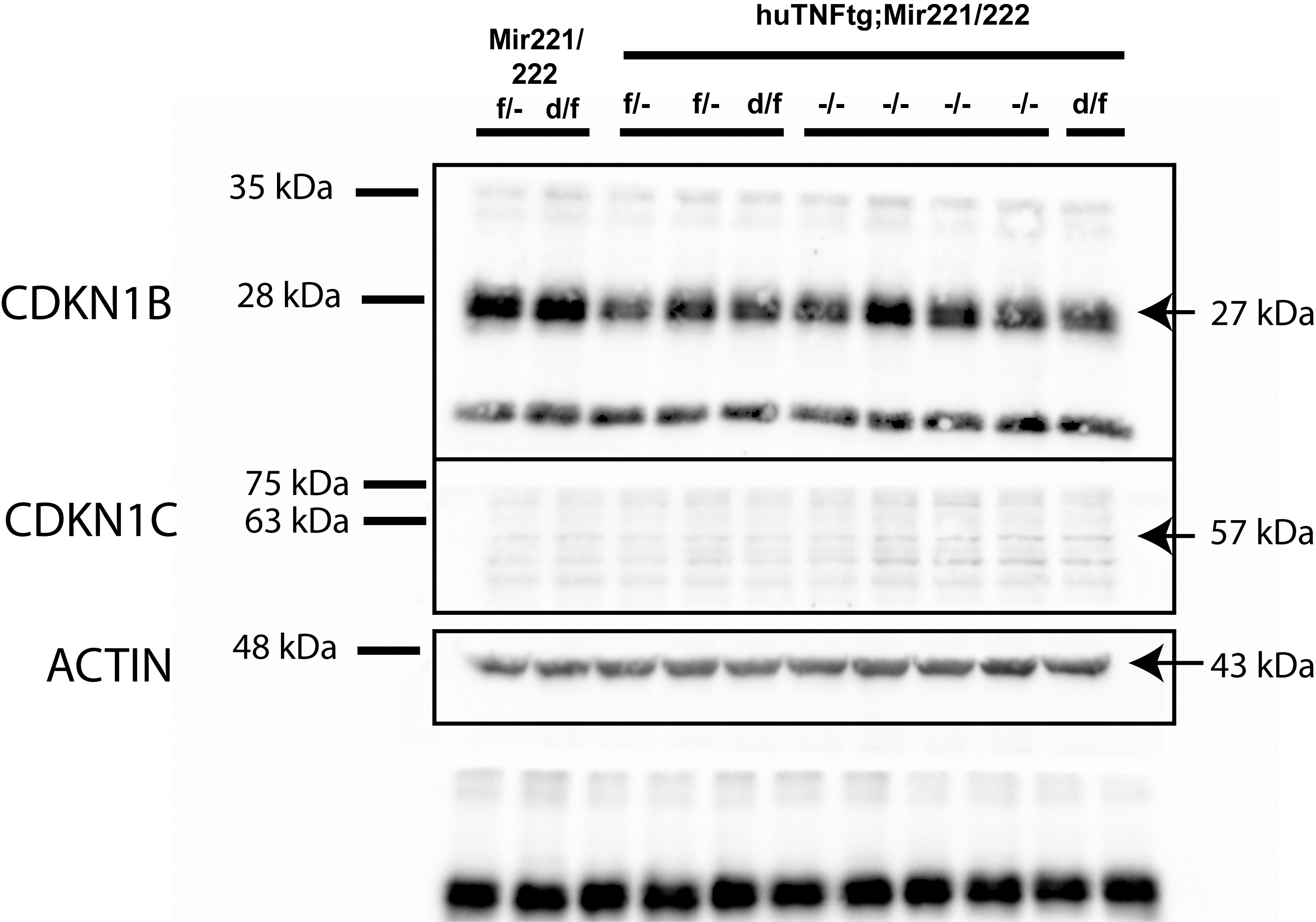

Supplement: Figure 5—source data 2. — Exposure set at 2.4 s for ACTIN. [file elife-84698-fig5-data2.zip › Figure 5-Source data 2.tif]

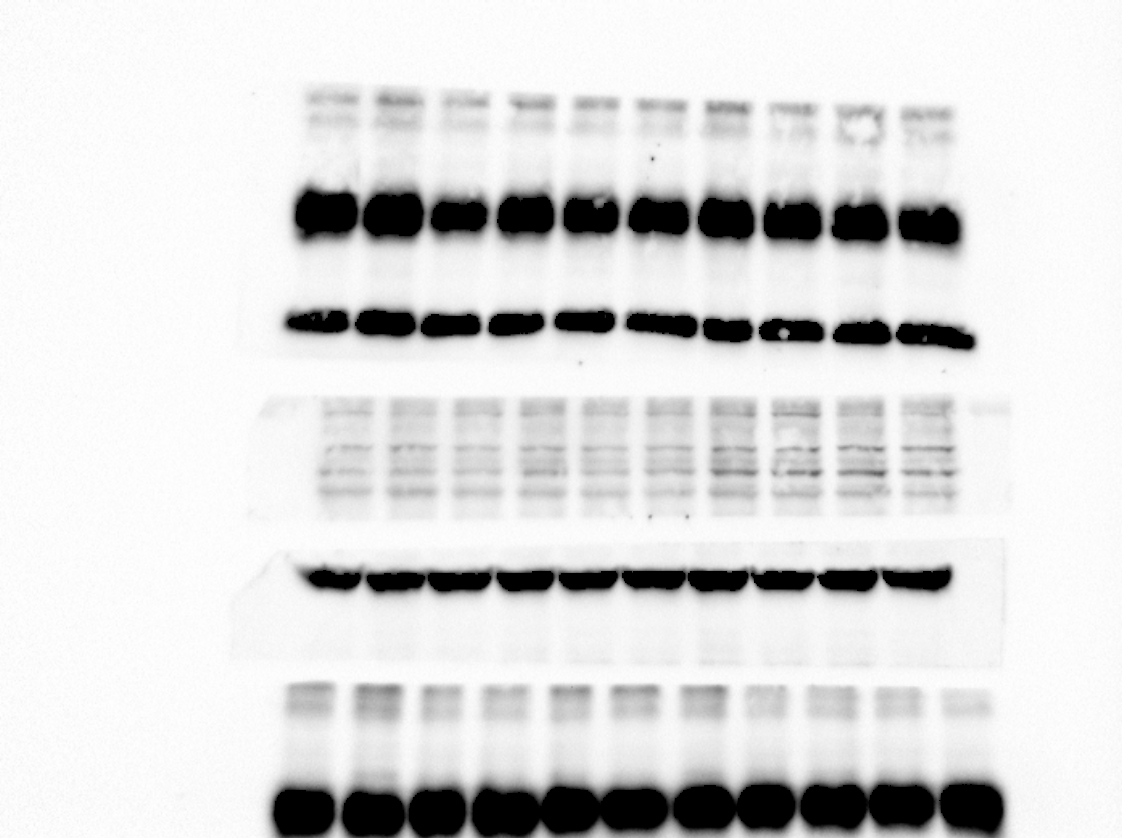

Supplement: Figure 5—source data 3. — Exposure set at 6.8 s for CDKN1C. [file elife-84698-fig5-data3.zip › Figure 5-Source data 3-raw-file.tif]

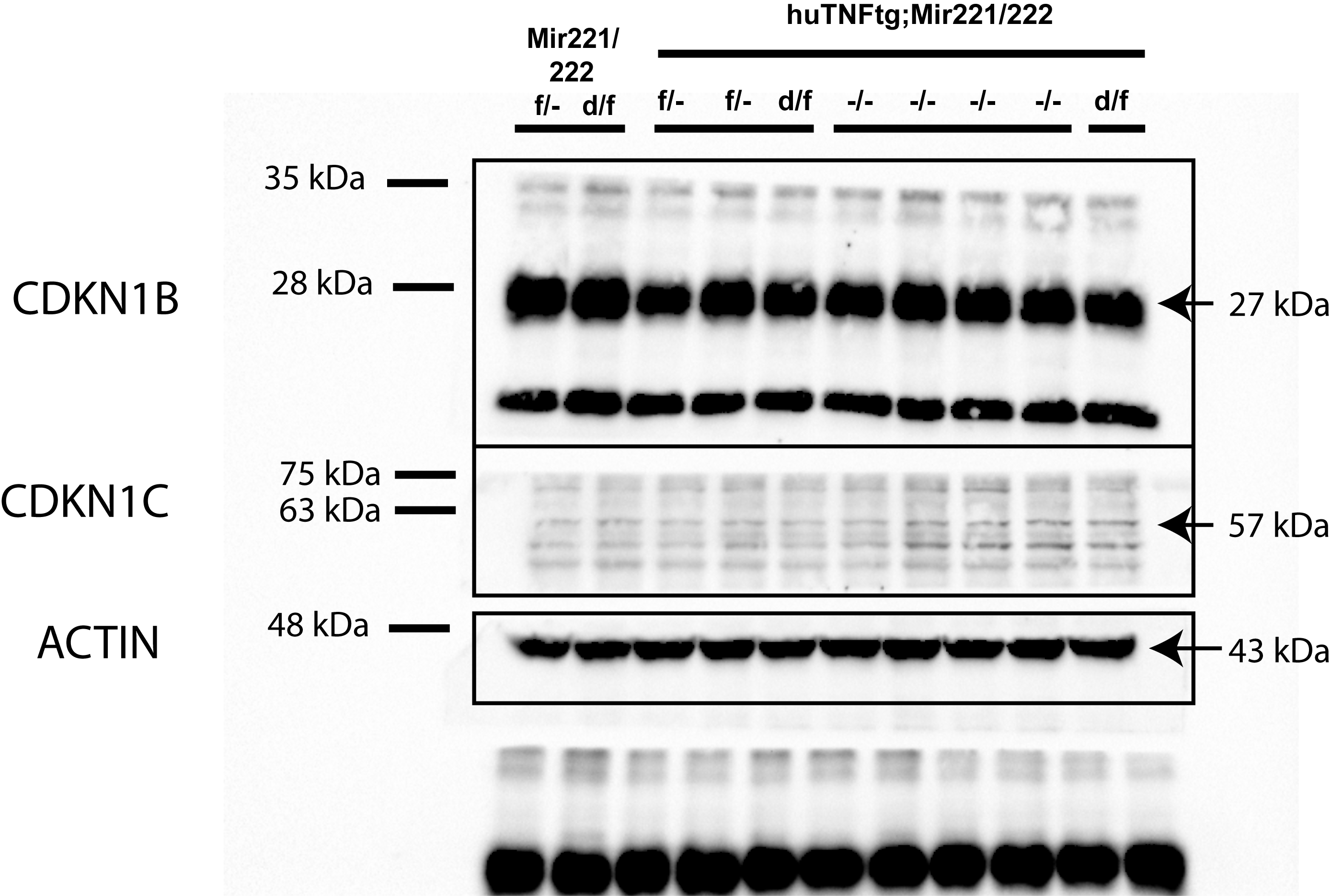

Supplement: Figure 5—source data 3. — Exposure set at 6.8 s for CDKN1C. [file elife-84698-fig5-data3.zip › Figure 5-Source data 3.tif]

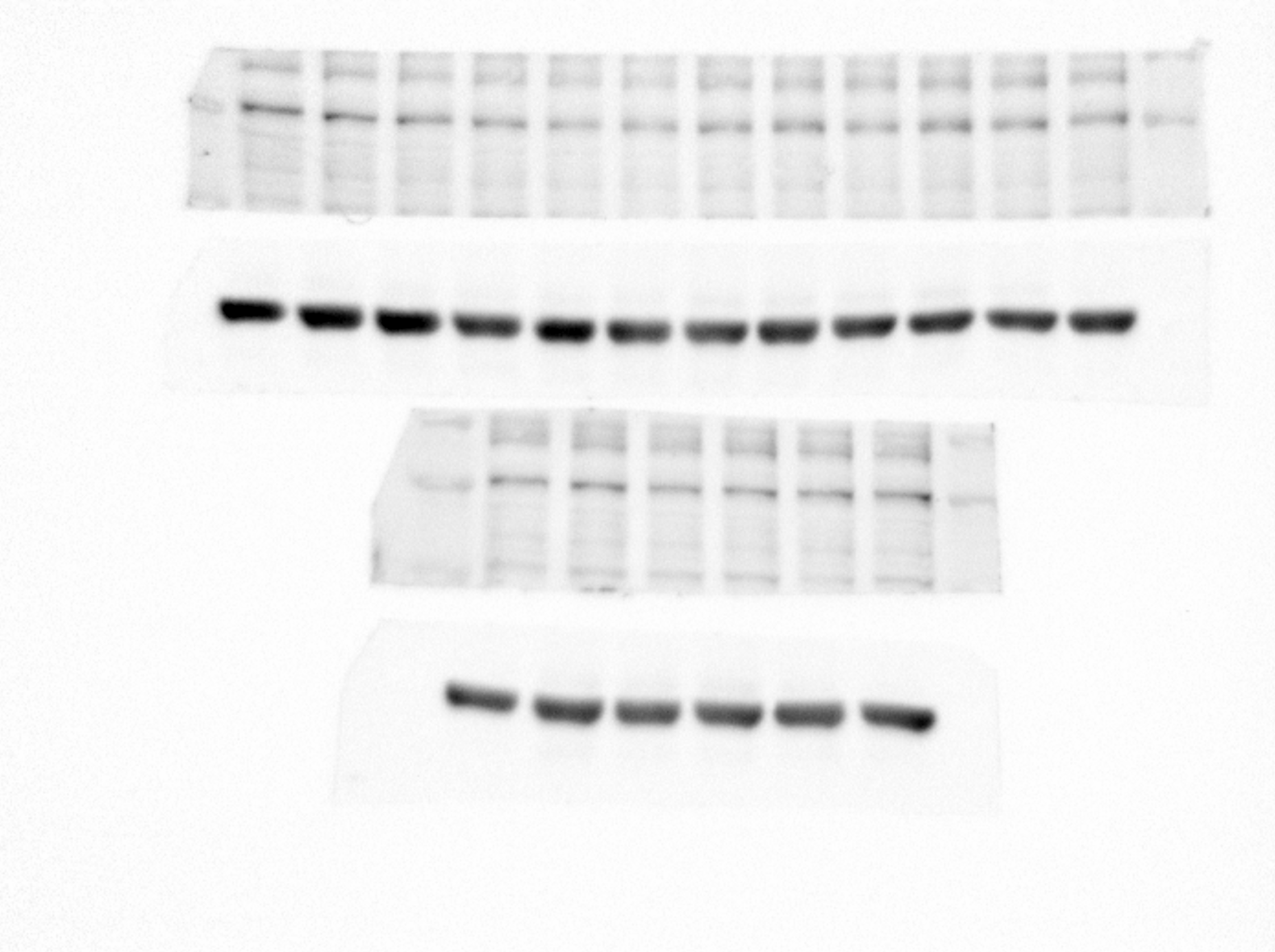

Supplement: Figure 5—source data 4. — Exposure set at 3.8 s for SMARCA1 and ACTIN. [file elife-84698-fig5-data4.zip › Figure 5-Source data 4 raw-file.tif]

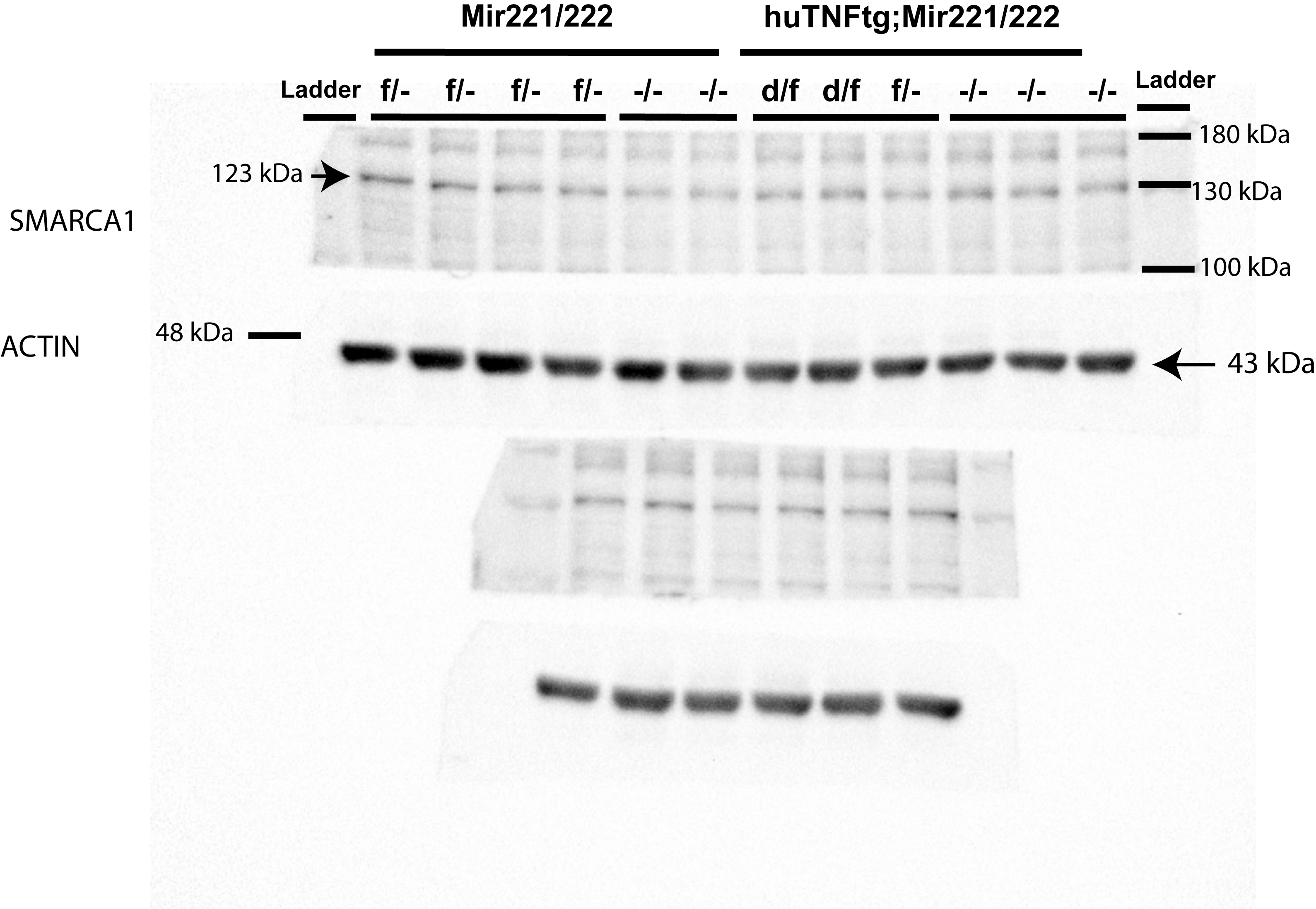

Supplement: Figure 5—source data 4. — Exposure set at 3.8 s for SMARCA1 and ACTIN. [file elife-84698-fig5-data4.zip › Figure 5-Source data 4.tif]
