# Supplementary material for: Mir221/222 drive synovial hyperplasia and arthritis by targeting cell cycle inhibitors and chromatin remodeling components
Source: eLife. 2024 Sep 5;13:e84698. doi: 10.7554/eLife.84698 (PMC11377061; doi:10.7554/eLife.84698)
Supplement: Supplementary file 1. [file elife-84698-supp1.docx]

**SUPPLEMENTARY FILE 1**

Supplementary file 1a: **Sequence of the PCR primers used for mouse genotyping in the study**

| huTNFtg 1 | 5'-TACCCCCTCCTTCAGACACC-3' |
| --- | --- |
| huTNFtg 2 | 5'-GCCCTTCATAATATCCCCCA-3' |
| TgCol6a1-Mir221/222 1 | 5'-TCAGCTCTGGGCTCTGACT-3’ |
| TgCol6a1-Mir221/222 2 | 5’-ACTGGGGCATATGATC TTGTC-3’ |
| Mir221/222 ^-/-^ 1 | 5'-GAGACTCTGGCTACTCATCC-3' |
| Mir221/222 ^-/-^ 2 | 5'-CCTTCAGCAAGAGCTGGGGAC-3' |
| Mir221/222 ^-/-^ 3 | 5'-GCTCTGTTTTCCTAAGTGATGG-3' |
| Mir221/222 ^-/-^ 4 | 5'-CTGACAGGAAGTAAATCATCTTAGC-3' |
| Cre 1 | 5'-ATTACCGGTCGATGCAACGAGT-3' |
| Cre 2 | 5'-CAGGTATCTCTGACCAGAGTCA-3' |
| Tnfr1 ^-/-^ 1 | 5'-CTGAATGAACTGCAGGACGA-3' |
| Tnfr1 ^-/-^ 2 | 5'-ATACTTTCTCGCCAGGAGCA-3' |
| Tnfr1 ^-/-^ 3 | 5'-CTGGAAGTGTGTCTCACTCAG-3' |
| Tnfr1 ^-/-^ 4 | 5'-TGCAGGGAGTGTGAAAAGG-3' |

Supplementary file 1b: **Sequence of the qPCR primers used in the study**

| *Cdkn1b* Forward | 5’-TCGACGCCAGACGTAAACAG-3′ |
| --- | --- |
| *Cdkn1b* Reverse | 5’-TTCAATGGAGTCAGCGATATGT-3′ |
| *Cdkn1c* Forward | 5’-CAATCAGCCAGCAGAACAGC-3′ |
| *Cdkn1c* Reverse | 5’-CAGCTCCTCGTGGTCTACAG-3′ |
| *Smarca1* Forward | 5’-GGGTTCCATCTCTCCGTGTT-3′ |
| *Smarca1* Reverse | 5’- AGTCACGCAAACATCCCACT-3′ |
| *Prg4* Forward | 5’-ATGGTAAGCCAGTGGATGGAC-3′ |
| *Prg4* Reverse | 5’-CGGTAATTCTGCGTGGTGGA-3′ |
| *Itga3* Forward | 5’-GAGCTGTGGTTGGTGCTTG-3′ |
| *Itga3* Reverse | 5’-GCACTTCCACAAGAGGAGGAT-3′ |
| *Ccl2* Forward | 5’-CCCAAAGAAGCTGTAGTTTTTGTCA-3′ |
| *Ccl2* Reverse | 5’-ACCCATTCCTTCTTGGGGTC-3′ |
| *Cxcl9* Forward | 5’-CGAGGCACGATCCACTACAA-3′ |
| *Cxcl9* Reverse | 5’-AGGCAGGTTTGATCTCCGTT-3′ |
| *Il6* Forward | 5’-GTTCTCTGGGAAATCGTGGA-3′ |
| *Il6* Reverse | 5’-TCCAGTTTGGTAGCATCCATC-3′ |
| *TNF* Forward | 5’-CTTCTCGAACCCCGAGTGAC-3′ |
| *TNF* Reverse | 5’-CCCTTGAAGAGGACCTGGGA-3′ |
| *Mmp3* Forward | 5’-GTCTCCCTGCAACCGTGAA-3′ |
| *Mmp3* Reverse | 5’-CCACCCTTGAGTCAACACCT-3′ |
| *Rankl* Forward | 5'-TCAGAAGACAGCACTCACTGC-3' |
| *Rankl* Reverse | 5'-CATTGATGGTGAGGTGTGCA-3' |
